# Fresh Meat from New Zealand

**Published:** 1883-01

**Authors:** 


### FRESH MEAT FROM NEW ZEALAND.

The sailing vessel Dunedin, belonging to the Albion Shipping Company, lately arrived in the East India Docks, London, with the first consignment of frozen meat which has been sent to England from New Zealand. This shipment differs from all other importations of frozen meat, from the fact of having been made in a sailing vessel, which has been 98 days on the passage, during which time the holds of the ship containing the meat have been kept at about 20° below freezing point. The vessel has on board 5,000 sheep, and the apparatus for freezing was fitted up by the Bell-Coleman Mechanical Refrigerating Company (limited).

The meat was in fine condition, and the shipment has been managed by the New Zealand and Australian Land Company (limited).

The success of this refrigerating sailing vessel ought to lead to a great extension of the trade in tropical fruits between New York and the West Indies. By the use of a refrigerating machine the immense losses now experienced by our fruit ships may be wholly overcome, and the finest fruits may be delivered here in prime condition. Vegetables may also be brought from the South without loss.

